# Synthesis of novel conjugated benzofuran-triazine derivatives: Antimicrobial and in-silico molecular docking studies

**DOI:** 10.1016/j.heliyon.2023.e18759

**Published:** 2023-07-27

**Authors:** Zahra Riyahi, Parvin Asadi, Farshid Hassanzadeh, Elahe Khodamoradi, Alexa Gonzalez, Mahmood Karimi Abdolmaleki

**Affiliations:** a Department of Chemistry, Shahreza Branch, Islamic Azad University, P.O. Box 311-86145, Shahreza, Isfahan, Iran; b Department of Medicinal Chemistry, School of Pharmacy and Pharmaceutical Sciences, Isfahan University of Medical Sciences, Isfahan, 81746-73461, Iran; c Isfahan Pharmaceutical Sciences Research Center, Isfahan University of Medical Sciences, Isfahan, Iran; d Department of Pharmaceutical Biotechnology, School of Pharmacy and Pharmaceutical Sciences, Isfahan University of Medical Sciences, Isfahan, Iran; e Department of Nursing, Texas A&M International University, Laredo, TX 78041, USA; f Department of Physical and Environmental Sciences, Texas A&M University-Corpus Christi, 6300 Ocean Drive, Corpus Christi, TX 78412, USA

**Keywords:** Anti-bacterial agent, Benzofuran, Triazine, In-silico study

## Abstract

Two new developments of antibacterial agents, a series of benzofuran-triazine based compounds (**8a-8h**) were designed and synthesized. The derivatives were prepared through conventional chemical reactions and structurally characterized with FT-IR, ^1^H and ^13^C NMR techniques. The antibacterial activity of the synthesized derivatives was assessed against gram-positive bacterial strains *(Bacillus subtilis,* and *Staphylococcus aureus*) and gram-negative bacterial strains (*Salmonella entritidis* and *Escherichia coli*). Compound **8e**, with the MIC value of 125-32 μg/μl against all the examined strains of bacteria, was the most active antibacterial compound. The synthesized derivatives were also studied for docking to the binding sites of *dihydrofolate reductase (DHFR)* receptor which has a key role in drug resistance associated with bacterial infections. The synthesized compounds showed good interaction with the targets through hydrogen bonding and hydrophobic interactions. According to antibacterial and docking studies, compound **8e** could be introduced as a candidate for development of antibacterial compounds.

## 1 Introduction

Recently, numerous biologically active compounds have been designed and synthesized through the hybridization of different drug pharmacophores and introduced as new therapeutic agents with extensive pharmacological activities [[1], [2], [3]]. In this method, it is claimed that the pharmacophores used in the structure of the compounds can be a powerful center for biological activity. There are many studies in which hybridization is used to strengthen the biological effect of two pharmacophores possessing the same biological effects [4,5]. Many of the presented biologically active hybrid molecules are under different phases of clinical trials [[6], [7], [8]].

Replacing three carbons of a benzene ring with nitrogen resulted in a triazine structure with different isomers according to the placement of the nitrogen atoms. 1,3,5-triazine derivatives possess promising *in vitro* and *in vivo* biological activity including, but not limited to, antiviral, anticancer, anti-inflammatory, antimicrobial, analgesic, antiprotozoal, antimalarial, antiproliferative, anti-depressant, and anti-fungal activity, which allows sequential introduction of this heterocyclic into the biologically active compounds [[9], [10], [11]]. Molecular hybridization between the triazine subunit and another antibacterial pharmacophoric heterocyclic leads to antimycobacterial compounds with activity against gram-positive and gram-negative bacteria. For example, novel *chalcone-triazine hybrids* and corresponding Cu-complex demonstrated an outstanding antibacterial effect on *S. aureus* and *E. coli* [12]. In another study, by Mekheimer et al., tetrazole-triazine hybrids were introduced as potent antifungal and antibacterial compounds [13]. Additionally, hybridization of 1,3,5-triazine with 4-aminoquinoline resulted in a compound which showed potent activity on *P. vulgaris*, *P. aeruginosa* and *S. aureus* [14]. It was also indicated that the presence of lipophilicity and aromaticity could increase the antibacterial activity of triazine derivatives [15].

Benzofuran as an oxygen-containing heterocyclic involving a fused benzene and furan ring scaffold has presented a wide range of pharmaceutical properties [16]. Benzofuran, as an important constituent of antibacterial natural products [17] could exert antibacterial activity through various mechanisms [18,19]. Due to its pronounced antibacterial activity, the use of benzofuran natural compounds in combination with other heterocyclics to produce novel antibacterial hybrid compounds has caught the interest of medicinal chemists. Benzofuran has shown good antibacterial effects as a hybrid with triazole [20] and isatin [21], pyrazole [22], thiazole [21], oxadiazoles [23], quinazolines [24], and pyrimidine [25].

**Scheme 1:**
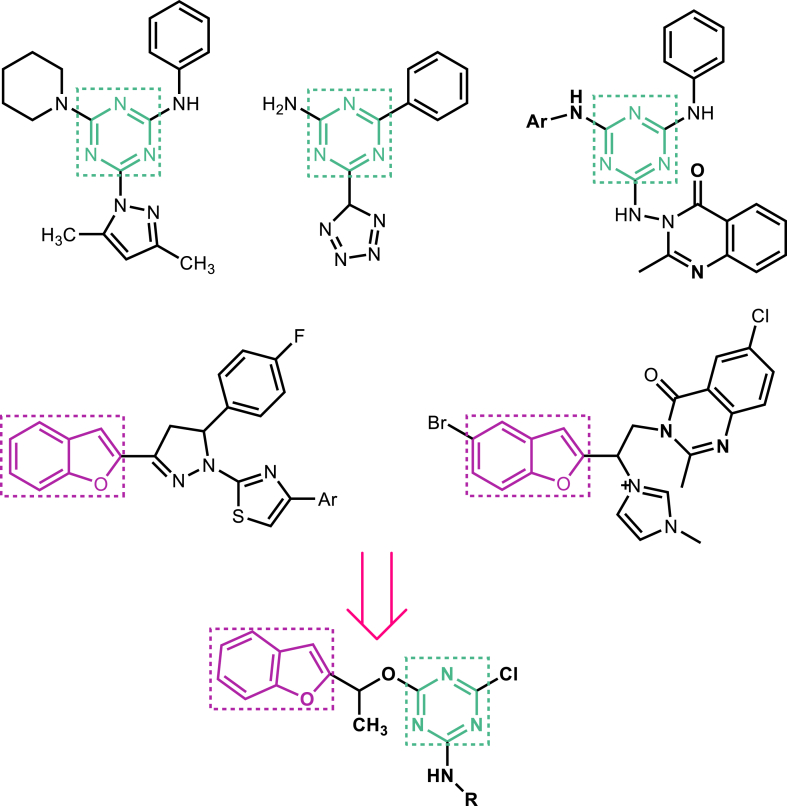
Design of novel benzofuran-triazine hybrids.

In the extension of our research on biologically active heterocyclics [11,19,24] and in consideration of the structure of numerous antibacterial hybrid compounds based on triazine [[12], [13], [14], [15]] and benzofuran, some of the new triazine and benzofuran hybrid derivatives were designed, synthesized, and evaluated for antibacterial activities (Scheme 1). Our strategy hypothesized that hybridization of 1,3,5-triazine with benzofuran may provide valuable therapeutic intervention for bacterial infections. The novel synthesized hybrids (8a-8h) were investigated for *in vitro* antibacterial activity against gram-positive *(Staphylococcus aureus and Bacillus subtilis*) and gram-negative (*Escherichia coli* and *Salmonella entritidis*) bacterial strains through the Microplate Alamar Blue Assay (MABA) method. Moreover, the binding mode of these newly hybrid derivatives was studied for *in silico* inhibition of the bacterial DHFR enzyme.

## 2 Experimental

### 2.1 Material and method

All of the reagents used for synthesis of the novel hybrid compound were synthetic grade and purchased from known Aldrich and Merck companies. The solvent was obtained from Merck Company. The synthesized compounds were characterized with different spectroscopic techniques. KBr sample plates were used to obtain Infrared spectra by Jasco-680 FT-IR (Japan) spectrometer (FT-IR). Nuclear magnetic resonance (NMR) Spectrophotometer (Bruker 500 MHz, Germany) was utilized to record proton and carbon (^1^H NMR & ^13^CNMR) spectra in DMSO‑*d*_6_ with trimethyl silane as the internal reference. To obtain melting points of newly synthesized derivatives and to confirm the purity of the starting material, an electrothermal 9200 melting point apparatus (UK) was used. Additionally, silica gel 60 F254 plates (Merck, Germany) were applied in thin-layer chromatography (TLC) to assess the purity of the compounds and evaluate the reaction progression.

### 2.2 Preparation of benzofuran-triazine hybrids

**Scheme 2:**
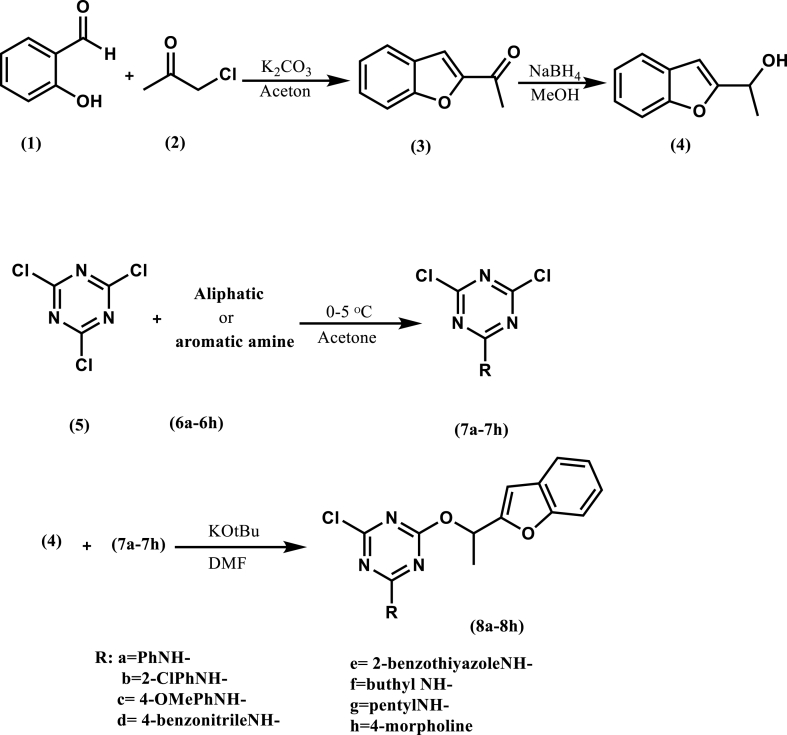
Synthetic rout of benzofuran-triazine hybrid derivatives.

The desired derivatives were prepared as schematic methods represented in Scheme 2.

#### 2.2.1 Preparation of 1-(benzofuran-2-yl)ethan-1-one (3)

Anhydrous potassium carbonate (6.0 mmol) was added to the dissolved salicylaldehyde (**1**, 3.0 mmol) in acetone (12 mL). Chloroacetone (**2**, 3.2 mmol) was added to the above mixture after a fine yellow color was formed in the reaction medium, which results from the removal of acidic hydrogen from salicylaldehyde. The obtained blend was heated under reflux condition for 5 h. The end of reaction was confirmed by TLC, then the mixture reaction was cooled to room temperature and filtered to remove the K_2_CO_3_. The obtained clear solution was filtered by vacuum to yield the desired product (**3**) which was then recrystallized in chloroform (yield: 90%) [24].

#### 2.2.2 Preparation of 1-(benzofuran-2-yl)ethan-1-ol (4)

Sodium borohydride (3.00 mmol) was gradually added to the solution of 1-(benzofuran-2-yl)ethan-1-one (3, 3.00 mmol) in methanol (10.00 mL) at 0 °C. The mixture was stirred and allowed to reach room temperature. The reaction then continued at this temperature for 5 h until TLC confirmed the reaction had been completed. Finally, diluted HCl (1:5, 3 mL) was added to the reaction mixture and the desired crude product was extracted with chloroform (yield: 65%) [26].

#### 2.2.3 Preparation of 4,6-dichloro-1,3,5-triazin-2-yl derivatives (7a-7h)

Dichloro-substituated triazine derivatives **(7a-7h)** were synthesized through a reaction of 5 mmol of different amine compounds with cyanuric chloride (**5**, 5 mmol) in acetone (10 mL) at 0–5 °C. After completion of the reactions (confirmed by TLC), the solid products in the mixture reaction were filtered, washed with acetone, and recrystallized from heptane (yields: 7a = 95%, 7b = 90%, 7c = 78%, 7d = 80%, 7e = 82%, 7f = 90%, 7g = 88%, and 7h = 75% [11].

#### 2.2.4 Preparation of 4-(1-(benzofuran-2-yl)ethoxy)-6-chloro-1,3,5-triazin-2-yl derivatives (8a-8h)

For the synthesis of desired triazine-benzofuran hybrids, 3 mmol of synthesized dichlorotriazine derivatives (7a-7h) reacted with compound 4 (3 mmol) in 15 mL of THF containing *tert*-butyl potassium oxide (3 mmol). The reaction was stirred at R.T. for 1 h and then continued at 60 °C for 6–8 h. At the end of the reaction, confirmed by TLC, the mixture was filtered by vacuum, and the contents were diluted with water. Then, it was extracted with dichloromethane and dried over anhydrous Na_2_SO_4_. The desired precipitate was obtained by removing the solvent and recrystallized from ethanol.

4-(1-(Benzofuran-2-yl)ethoxy)-6-chloro-N-phenyl-1,3,5-triazin-2-amine (8a)

C_19_H_15_ClN_4_O_2_; Yield: 68%, White solid, mp: 280 °C, FT-IR (Cm^−1^), 3354 (N–H), 3117(aromatic C–H) 2922 (aliphatic C–H), 1675 (C=N), 1518, 1555, 1569 (aromatic C=C), 1320 (C–O), 771.7 (C–Cl). H NMR: (ppm): 1.71(3H, d, CH_3_, J = 6.54), 5 (1H, br, N–H), 5.7(1H, m. C–H, J = 5.2), 6.81 (1H, t, Ar, J = 7.57), 7 (1H, m, Ar), 7.2(2H, m, Ar), 7.3(1H, m, Ar), 7.4 (1H, m, Ar), 7.64 (2H, m, Ar), 7.9 (1H, m, Ar), 8.0 (1H, m, Ar).

4-(1-(Benzofuran-2-yl)ethoxy)-6-chloro-N-(2-chlorophenyl)-1,3,5-triazin-2-amine (8b)

C_19_H_14_Cl_2_N_4_O_2_; Yield: 65%, White solid, mp: 220 °C, FT-IR (Cm^−1^), 3378 (N–H), 3109(aromatic C–H), 2823 (aliphatic C–H), 1665 (C=N), 1557, 1538(aromatic C=C), 1325 (C–O), 785(C–Cl). H NMR: (ppm): 1.7 (3H, d, CH_3_, J = 6.6), 5(1H, br, N–H), 5.7 (1H, q. C–H, J = 5.2), 6.81(1H, m, Ar), 7 (1H, d, Ar, J = 8.2), 7.3(1H, m, Ar), 7.38(1H, m, Ar, J = 7.9), 7.4 (1H, m, Ar), 7.45(1H, m, Ar), 7.68 (1H, m, Ar), 7.7(1H, m, Ar),8 (1H, m, Ar).

4-(1-(Benzofuran-2-yl)ethoxy)-6-chloro-N-(4-methoxyphenyl)-1,3,5-triazin-2-amine (8c)

C_20_H_17_ClN_4_O_3_; Yield: 70%, White solid, mp: 226 °C, FT-IR (Cm^−1^), 3414 (N–H), 3124 (aromatic C–H) 2933 (aliphatic C–H), 1662(C=N), 1505 (aromatic C=C), 1320 (C–O), 763(C–Cl); ^1^H NMR: (ppm): 1.7 (3H, d, CH3, *J=*6.54), 3.6(3H,s, OCH3,), 4.9(1H, br,N–H), 5.7(1H, q, CH, *J=*5.23), 6.7(1H, t, Ar, *J=*7.57), 6.8(2H, m, Ar), 7.4(2H, m, Ar), 7.6(4H, m, Ar),

4-((4-(1-(Benzofuran-2-yl)ethoxy)-6-chloro-1,3,5-triazin-2-yl)amino)benzonitrile (8d)

C_20_H_14_ClN_5_O_2_S, Yield: 60%, White solid, mp: 265 °C, FT-IR (Cm^−1^), 3250 (N–H), 3109 (aromatic C–H), 2918 (aliphatic C–H), 2260 (C=N), 1669 (C=N), 1525, 1543, 1574 (aromatic C=C), 1325 (C–O), 780 (C–Cl). ^1^H NMR (ppm): 1.73 (3H, d, CH_3_, *J* = 6.59), 5.6 (1H, br, N–H), 5.8 (1H, m, C–H), 6.79(1H, t, Ar, J = 7.57), 7.4 (2H, m, Ar), 7.64 (2H, m, Ar), 7.7 (2H, d, Ar, *J* = 7.89), 7.9 (2H, d, Ar, *J* = 7.89), C_20_H_14_ClN_5_O_2_S (*m*/*z* = 423.5).

N-(4-(1-(benzofuran-2-yl)ethoxy)-6-chloro-1,3,5-triazin-2-yl)benzo [d]thiazol-2-amine (8e)

C_20_H_14_ClN_5_O_2_S, Yield: 60%, White solid, mp: 173 °C, FT-IR (cm^−1^), 3225 (N–H), 3099 (aromatic C–H), 2920 (aliphatic C–H), 1675 (C=N), 1597, 1566, 1587 (aromatic C=C), 1322(C–O), 750 (C–Cl). H NMR (ppm): 1.77 (3H, d, CH_3_, J = 6.59), 5.1 (1H,br, N–H), 5.8 (1H, m. C–H), 6.73 (1H, t, Ar, J = 7.60), 7.41 (2H, m, Ar), 7.59 (2H, m, Ar), 7.7 (2H, m, Ar), 7.9 (1H, t, Ar, J = 7.97), 8.1 (1H, m, Ar); ^13^C NMR: (DMSO‑*d*_6_, 100 MHz):19.3, 55.8, 100.0, 102.0, 118.3, 120.7, 121.8, 123.8, 124.1, 124.8, 125.9, 126.3, 130.2, 152.2, 154.7, 160.3, 168.8, 167.3, 177.6, and 178.4.

4-(1-(Benzofuran-2-yl)ethoxy)-N-butyl-6-chloro-1,3,5-triazin-2-amine (8f)

C_16_H_7_ClN_4_O_2_; Yield: 90%, White solid, mp: 115 °C, FT-IR (Cm^−1^), 3275 (N–H), 3093 (aromatic C–H) 2965 (aliphatic C–H), 1635(C=N), 1407 (aromatic C=C), 1323 (C–O), 766 (C–Cl). ^1^H NMR: (ppm): 0.8 (3H, t, CH_3_, J = 3.4), 1.4 (4H, m, C–H, aliphatic), 1.5(3H, d. C–H, J = 4.97), 3.3 (2H, m, C–H), 4.3 (1H, br, N–H,), 4.8 (1H, m, C–H, J = 5.96), 6.7(1H, m, Ar), 7.4 (2H, m, Ar), 7.7(2H, m, Ar).

4-(1-(Benzofuran-2-yl)ethoxy)-6-chloro-N-pentyl-1,3,5-triazin-2-amine (8g)

C_17_H_19_Cl_2_N_4_O_2_; Yield: 72%, White solid, mp: 105 °C, FT-IR (Cm^−1^), 3261 (N–H), 3089 (aromatic C–H) 2930 (aliphatic C–H), 1665 (C=N), 1561, 1569 (aromatic C=C), 1320 (C–O), 751(C–Cl). ^1^H NMR: (ppm): 0.89 (3H, t, CH_3_, *J* = 4.12), 1.2 (4H, m, C–H, aliphatic), 1.4 (2H, m, C-Hm), 1.6(3H, d, CH_3_, *J* = 6.12), 3.3 (2H, m, C–H,aliphatic), 4.8 (1H, br, N–H), 5.5 (1H, m, CH), 6.7 (1H, t, Ar, *J* = 17.68), 7.3 (2H, m, Ar,), 7.5(2H, m, Ar),.

4-(4-(1-(Benzofuran-2-yl)ethoxy)-6-chloro-1,3,5-triazin-2-yl)morpholine (8h)

C_17_H_17_ClN_4_O_3_; Yield: 65%, White solid, mp: 136 °C, FT-IR (Cm^−1^), 3243 (N–H), 3112 (aromatic C–H), 2927 (aliphatic C–H), 1645(C=N), 1412 (aromatic C=C), 1322 (C–O), 786 (C–Cl); H NMR: (ppm): 1.5 (3H, d. CH_3_, *J* = 4.97), 3.6(4H, t, CH_2_, *J* = 3.95), 3.9(4H, m, CH2, *J* = 3.95) 4.3(1H, br, N–H), 4.8 (1H, s, C–H), 6.7 (1H, s, Ar), 7.4 (2H, d, Ar, *J* = 8.00), 7.7 (2H, d, Ar, *J* = 8.00).

### 2.3 Antimicrobial activity

The synthesized benzofuran-triazine hybrid compounds (**8a-6h**) were subjected for antimicrobial study against gram-positive *(Staphylococcus aureus* and *Bacillus subtilis,*) and gram-negative (*Escherichia coli* and *Salmonella enteritidis)* bacterial strains using the MABA test method [24], performed with 10 mL of colony suspensions in 96-well sterile plates. Bacterial cells with a concentration of 10^8^ CFU/mL (McFarland 0.5 standard) were diluted to 1:999 by Mueller Hinton Broth (MHB). Chloramphenicol and ampicillin were used as a positive control for gram-negative and gram-positive strain, respectively. MHB without inoculum and MHB with inoculum were used as control and negative control, respectively. Different concentrations of synthesized compounds (0.3 to 5 μg/mL) were prepared via serial dilution with dimethyl sulfoxide (below 1%) and then added to the plate. After that, Alamar blue (DAL 1100 Biosource; Invitrogen, CA, USA) was added to the wells, and the plates were incubated at 37 °C to investigate antibacterial activity for 24 h. The lowest concentration, which had the same color as positive controls, was recorded as the minimum inhibitory concentration (MICs) of compounds.

### 2.4 Molecular docking

Docking studies of the synthesized benzofuran-triazine hybrids (8a-8h) to crystallography structure of *S. aureus* DHFR (PDB ID: 2W9S) as a bacterial target were carried out using AutoDock 4.2 [11]. X-ray structure of *S. aureus* DHFR via trimethoprim as a co-crystallized ligand with 1.80 Å resolution was retrieved from protein data bank. For initial preparation, crystallographic water molecules and ligand were eliminated from crystal structures and then the polar hydrogen, kollman charge was calculated for desired target by AutoDockTools-1.5.6 version. ChemDrow 8.0 and HyperChem softwares were used for drawing the chemical structures and optimization of compounds, respectively. All the optimized structures were converted to required PDBQT file format by AutoDockTools and docked to the desired protein. The binding energy and main interactions of the ligands were theoretically examined. To check the correctness of the docking method, RMSD of cocrystal ligand was calculated by redocking, which confirmed the little difference between redocking and initial poses, indicating that the binding prediction in docking is valid.

## 3 Results and discussion

For the synthesis of benzofuranyl ethanol (3) intermediate, the synthesized benzofuranyl-methyl-ketones (1) was reduced with sodium borohydride in methanol and then the desired triazine-benzofuran hybrids were obtained as precipitates by reaction of equimolar of synthesized dichlorotriazine derivatives (7a-7h) with compound **4**. The novel hybrids derivatives **(8a-8h)** were characterized through FT-IR and NMR techniques.

### 3.1 Characterizations

**Fig. 1:**
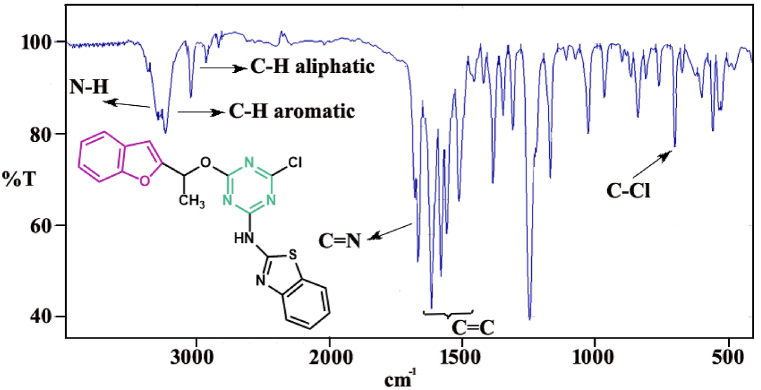
FT-IR spectrum of **8e** as a most potent antibacterial compound in the synthesized hybrids.

Essentially, assignments of the FT-IR spectral of the synthesized benzofuran-triazine derivatives confirmed the structure of compounds. The **8a-8h** compounds showed the absorptions at 3378-3225 cm^−1^ in their FT-IR spectral indicating the existence of N–H in the structures. The absorptions at 3112-3089 cm^−1^ were elated to stretching of aromatic C–H, and the peaks in 2965-2823 cm^−1^ confirmed the existence of aliphatic C–H. The absorptions in the region of 1400–1600 cm^−1^ are attributed to the aromatic moieties on the structures. The presence of a peak around 750 cm^−1^ is related to the C–Cl bond, which confirms that the third chlorine group remains on the triazine ring. In the FT-IR spectral of compound **8d** which has nitrile substitution, the presence of absorption at 2260 cm^−1^ indicates the existence of this group. FT-IR spectrum of **8e,** as the most potent antibacterial compound in this series, is shown in Fig. 1.

**Fig. 2:**
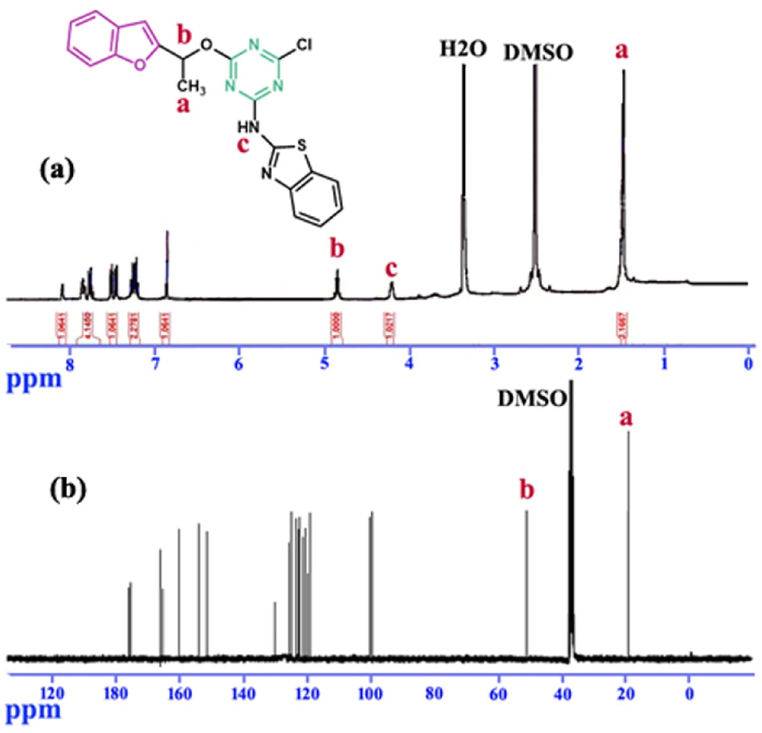
(a) ^1^H NMR and (b) ^13^CNMR spectra of the compound **8e** as a most potent antibacterial compound in the synthesized hybrids.

The ^1^H NMR spectrum of compound **8a-8h** showed absorptions around 1.51 ppm as a singlet peak which is related to methyl group adjacent to oxygen. Hydrogen of chiral centers appeared around 5.54 ppm as multiple peaks. The absorptions of aromatic hydrogens related to benzofuran and aromatic substitutions on triazine ring founded in the range of 6.85–7.95 ppm. A broad singlet peak at 4.5–5.5 ppm confirmed the presence of N–H group. The alkyl group of aliphatic substitutions is also seen in the area related to aliphatic hydrogens (Fig. 2a).

In ^13^C NMR spectrum of **8e** compound (Fig. 2b), the peaks 19.3 and 55.3 ppm are related to methyl and chiral center, respectively. Additionally, the presence of an adsorption of 15 in aromatic region of the spectrum supported the successful synthesis of the compound **8e**. ^1^H NMR and ^13^CNMR spectrum of compound **8e** as the most potent antibacterial compound in this series is shown in Fig. 2.

### 3.2 Antibacterial study

The synthesized hybrid compounds were subjected for antimicrobial study on gram-positive *(Staphylococcus aureus* and *Bacillus subtilis,*) and gram-negative (*Salmonella entritidis* and *Escherichia coli*) bacterial strains. The obtained results are represented in Table 1.

**Table 1:** The obtained MIC for the synthesized hybrids (**8a-8h**) on gram-positive and gram-negative bacteria.


		MIC (μg/ml)			
		Gram-positive		Gram-negative	
code	R	*Staphylococcus aureus*	Bacillus subtilis	Escherichia Coli	Salmonella entritidis
8a	phenyl	250	250	500	500
8b	2-chloro phenyl	125	125	500	500
8c	4-methoxy phenyl	250	125	500	250
8d	4- benzonitrile	125	125	125	125
8e	2-benzothiazole	32	125	32	32
8f	butyl	250	250	500	500
8g	pentyl	32	250	500	500
8h	morpholine	250	250	250	500
	Penicillin	16	16	8	8
	Chloramphenicol	4	4	16	16

The results of antimicrobial study on gram-negative and gram-positive bacteria exhibited that some synthesized benzofuran-triazine compounds (**8e, 8g, 8d**) showed acceptable antibacterial activities at the concentrations of 32–125 μg/mL in comparison with positive controls. Compound **8e** with amino benzothiazole moiety was the most potent antibacterial derivative, which showed IC_50_ = 32, 125, 32 and 32 μg/mL on *Escherichia coli*, *Bacillus subtilis*, *Staphylococcus aureus* and *Salmonella enteritidis*, respectively. In this compound, in addition to two antibacterial benzofuran and triazine pharmacophores, 2-aminoibenzothiazole as another antibacterial pharmacophore has also been used. In fact, the placement of a pharmacophore with proven antibacterial properties help to increase the antibacterial activity of the benzofuran-triazine hybrids.

The antimicrobial study also indicated that the synthesized compounds showed better antibacterial activity on gram-positive rather than gram-negative bacteria. Additionally, with the exception of **5g** with IC_50_ = 32 μg/mL on *Staphylococcus aureus*, the placement of aliphatic groups on the triazine ring showed a lower antibacterial effect than aromatic substitutions on both gram-negative and gram-positive bacteria.

### 3.3 Docking

**Fig. 3:**
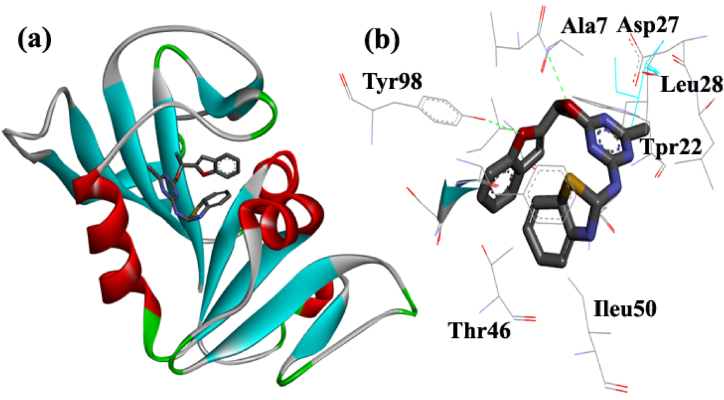
The binding mode (a) and interactions (b) of compound **8e** in the active site of DHFR.

Molecular docking, an important approach for modeling the atomic interaction between a small molecule and a protein, has become an increasingly important tool for drug discovery. In this study after the antibacterial study, the *in-silico* study was performed to theoretically predict the probable interaction of the derivatives on a significant bacterial target and examine their potential as a bacterial enzyme inhibitor. Since drug resistance associated with the DHFR enzyme has emerged as a critical issue in the treatment of bacterial infections, it was selected for the molecular docking study of ligands. The obtained docking results including binding energies, inhibition constant (Ki), hydrophobic interactions, and hydrogen bond were shown in Table 2. The binding mode and interactions of compound **8e** as the most antibacterial compound in the active site of DHFR was shown in Fig. 3a and b, respectively.

**Table 2:** Binding energy, inhibition constant (Ki) and interaction of synthesized benzofuran-triazine hybrids obtained from docking study.

Name of compound	Binding energy (kcal/mol)	Inhibition constant	H bond interaction	Hydrophobic interaction
8a	−8.56	150 nm	Phe92 Th46	Aniline: Phe92, Ileu31, val6
				Triazin: Ileu50, Phe92
				Benzofuran: Trp22, Ileu28, Asp27
8b	−8.61	160 nm	Not seen	Cl-Aniline: Thr14, Ileu14, Leu20
				Triazin: Phe92, Ileu31
				Benzofuran: Ileu50, Ser49
8c	−8.34	175 nm	Phe92	Anisidine: Tyr98, Gly15. Thr46
				Triazin: Ileu50, Phe92, Ileu5
				Benzofuran: Leu54, Leu28
8d	−8.20	170 nm	Phe92 Tyr98	Benzonitrile: Tyr98, Phe92. Thr46
				Triazin: Ileu50, Ileu5
				Benzofuran: Leu54, Val6
8e	−8.75	490 nm	Tyr98 Val7	Aminothiazole: Thr46, Ileu50, Leu20
				Triazine: Ileu14, Phe92
				Benzofuran: Trp22, Ileu28, Asp27
8f	−8.49	593 nm	Leu20, Ser49	Butyl: Asn18, Ileu14
				Triazin: Ileu50, Leu20
				Benzofuran: Phe92, Val6
8g	−8.95	275 nm	Tyr98, Phe92	Pentyl: Asp27, Ileu50, Leu20
				Triazin: Gln19, Gly15
				Benzofuran: Val6, Ileu5, Phe92
8h	−7.98	642 nm	Tyr98	morpholine: Tyr98, Ileu31, Ileu50
				Triazin: Val7, Phe92
				Benzofuran: Trp22, Ileu28,

All compounds have shown a binding energy of about −8 kcal/mol and the ability to form a hydrogen bond (except **8b**) with the desired target, which confirms that the synthesized compounds are theoretically capable of effective interactions. Comparison of biological results with docking studies results indicated that despite the good theoretical interaction of these compounds with the desired target in docking studies, some of these compounds have not shown good antibacterial effect. In explanation of the observed difference, it should be said that the docking study is just a theoretical study, and it does not examine the effective biological factors that are effective in attaching the compound to the enzyme. In addition, the antibacterial mechanism of these novel hybrids is still unknown. According to the obtained result, the selection of benzofuran and triazine pharmacophores could lead to the compounds among which compound **8e** can be selected as a good antibacterial compound and be considered for further studies.

## 4 Conclusions

In this work, novel hybrid heterocyclic derivatives, containing the benzofuran and triazine rings were designed, synthesized, and characterized by spectroscopic methods. The antibacterial activity of the derivatives was studied on gram-positive *(Staphylococcus aureus* and *Bacillus subtilis,*) and gram-negative (*Escherichia coli* and *Salmonella entritidis*) bacterial strains. The obtained results indicated that some compounds (**8e, 8d, 8g**) had good antimicrobial activity and compound **8e,** with the MIC value 32–125 μg/mL against all the experienced strains of microorganisms, identified as a most active derivatives in this group. All the derivatives were docked into the binding sites of DHFR, an enzyme which has a key role in drug resistance associated with bacterial infections. All the derivatives interact with DHFR through H-bond and hydrophobic interactions. Based on the results from the antibacterial and docking studies, compound **8e** could be introduced as a candidate for development of antibacterial compounds.

## Author contribution statement

Parvin Asadi; Mahmood Karimi Abdolmaleki: Conceived and designed the experiments.

Zahra Riyahia; Farshid Hassanzadeh: Performed the experiments.

Elahe Khodamoradi; Alexa Gonzalez; Zahra Riyahi: Analyzed and interpreted the data; Wrote the paper.

## Data availability statement

Data included in article/supp. material/referenced in article.

## Declaration of competing interest

The authors declare that they have no known competing financial interests or personal relationships that could have appeared to influence the work reported in this paper.
