# Supplementary material for: Synthesis of novel conjugated benzofuran-triazine derivatives: Antimicrobial and in-silico molecular docking studies
Source: Heliyon. 2023 Jul 27;9(8):e18759. doi: 10.1016/j.heliyon.2023.e18759 (PMC10412834; doi:10.1016/j.heliyon.2023.e18759)
Supplement: Multimedia component 1 [file mmc1.docx]

**Supplementary data**

**Synthesis of novel conjugated benzofuran-triazine derivatives: antimicrobial and in sillico molecular docking studies**

Zahra Riyahi^a^ , Parvin Asadi^*b,c^ , Farshid Hassanzadeh^b^ , Elahe Khodamoradi^d^ , Alexa Gonzalez^e^ , Mahmood Karimi Abdolmaleki^*f^

*^a^*) *Department of Chemistry, Shahreza Branch, Islamic Azad University, P.O. Box 311-86145, Shahreza, Isfahan, Iran*

*^b^*) *Department of Medicinal Chemistry, School of Pharmacy and Pharmaceutical Sciences, Isfahan University of Medical Sciences, Isfahan,* 81746-73461*, I.R.Iran*

*^c^) Isfahan Pharmaceutical Sciences Research Center, Isfahan University of Medical Sciences, Isfahan, Iran*

*E-mail: asadi@pharm.mui.ac.ir (P. Asadi)*

*^d^*) *Department of Pharmaceutical Biotechnology, School of Pharmacy and Pharmaceutical Sciences, Isfahan University of Medical Sciences, Isfahan, Iran*

*e) Department of Nursing, Texas A&M International University, Laredo, TX 78041, USA*

*f) Department of Physical and Environmental Sciences, Texas A&M University-Corpus Christi, 6300 Ocean Drive, Corpus Christi, TX 78412, USA*

^1^H NMR spectra……………………. Figure1-7


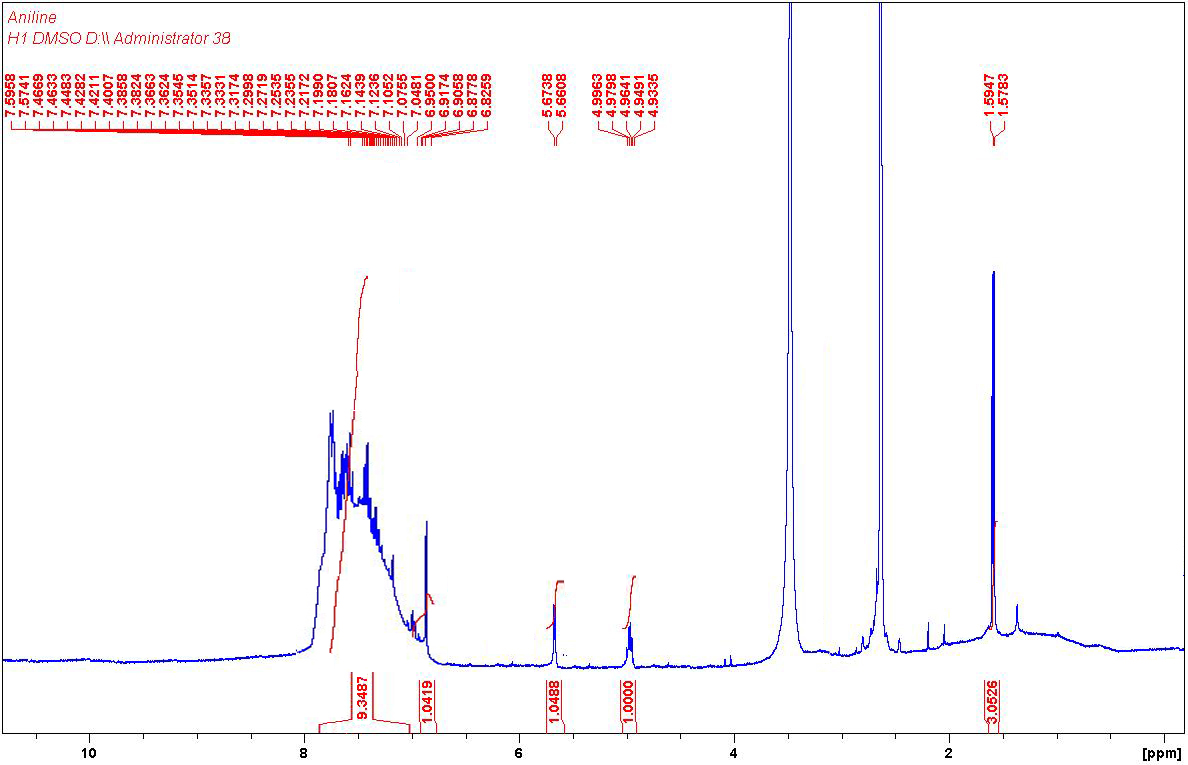


Figure 1. ^1^H NMR spectrum of compound (8a)


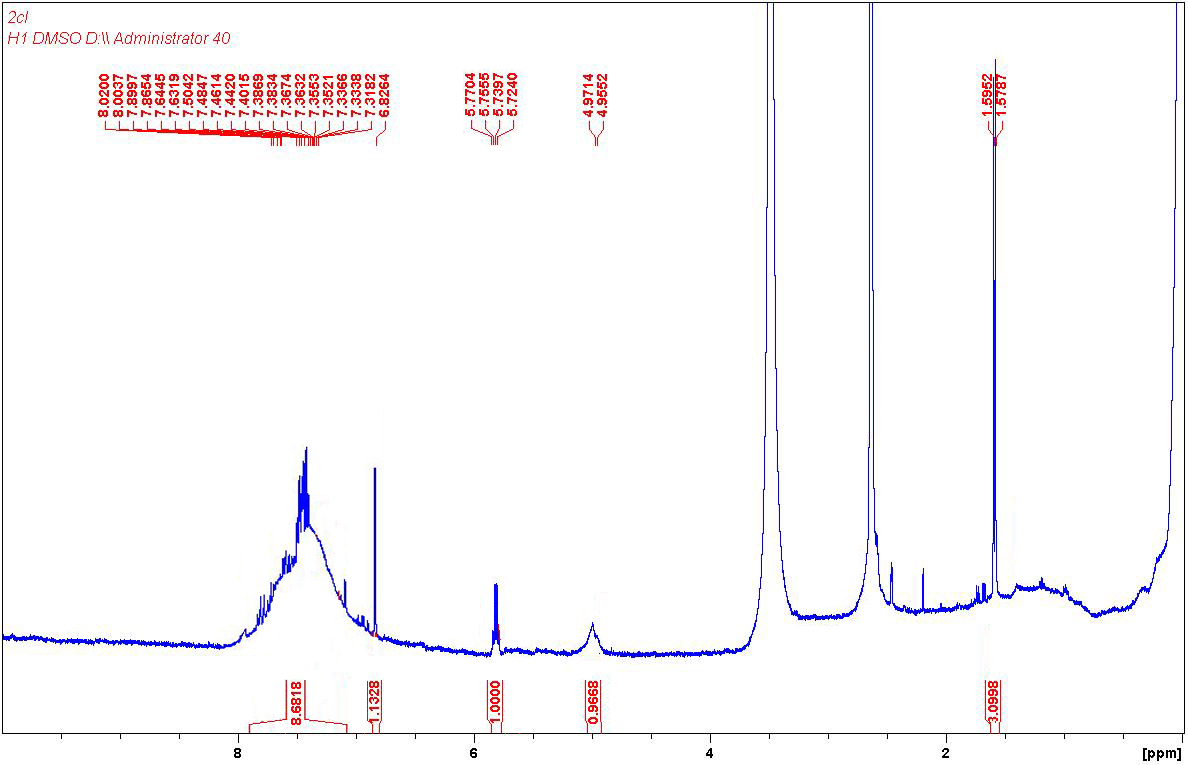


Figure 2. ^1^H NMR spectrum of compound (8b)


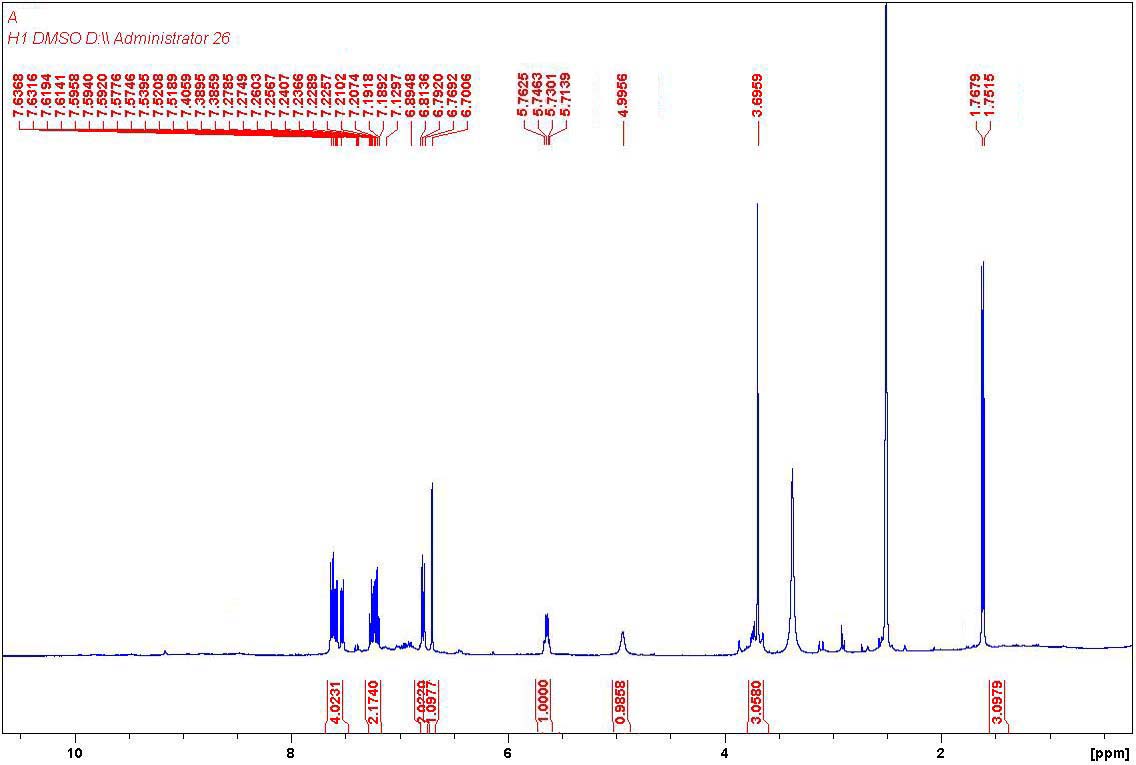


Figure 3. ^1^H NMR spectrum of compound (8c)


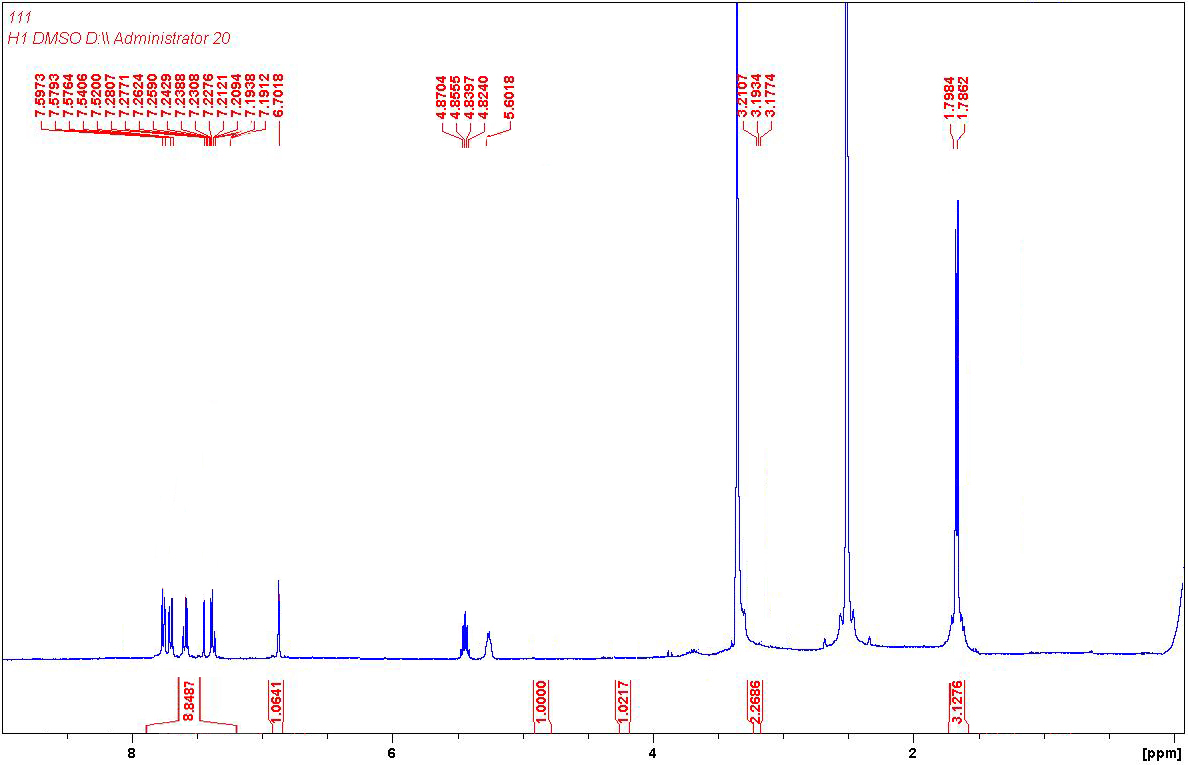


Figure 4. ^1^H NMR spectrum of compound (8d)


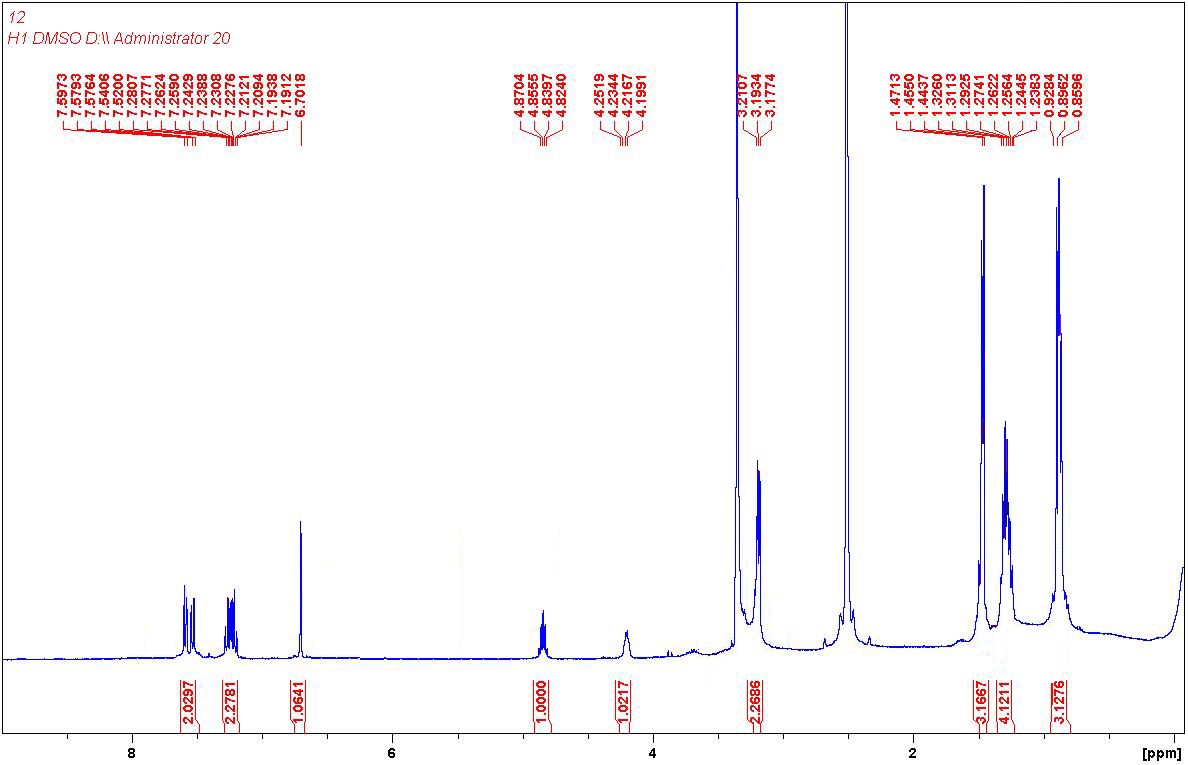


Figure 5. ^1^H NMR spectrum of compound (8f)


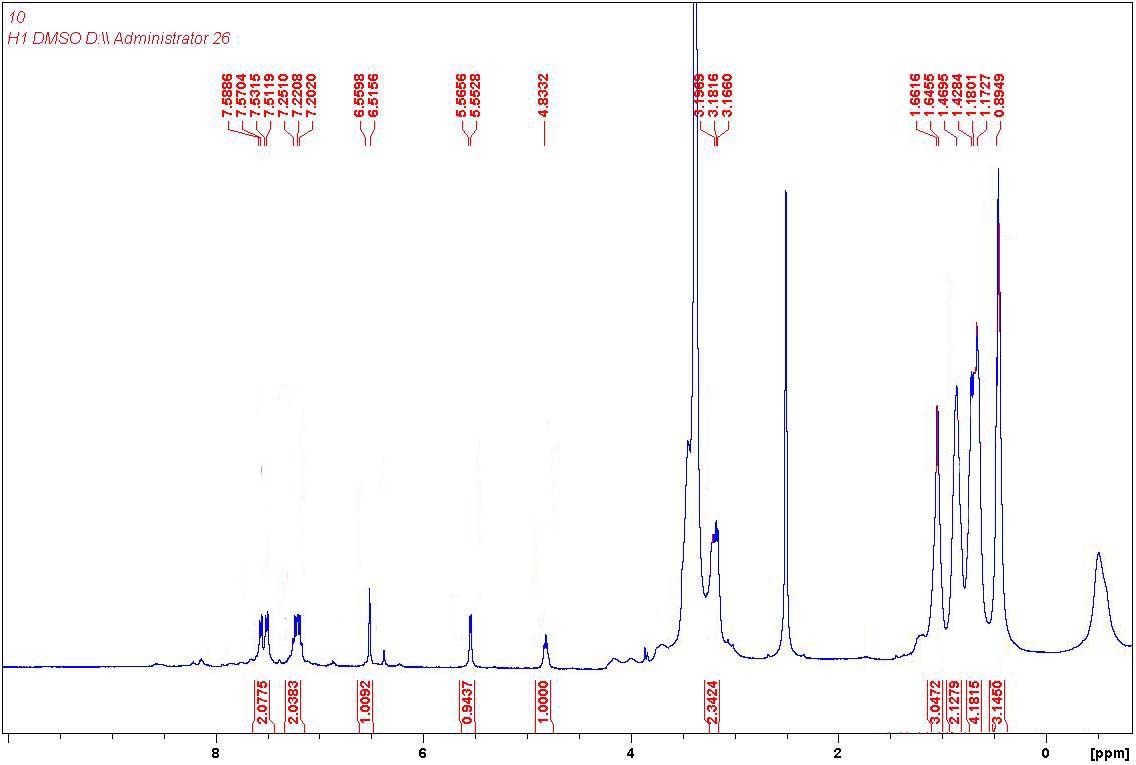


Figure 6. ^1^H NMR spectrum of compound (8g)


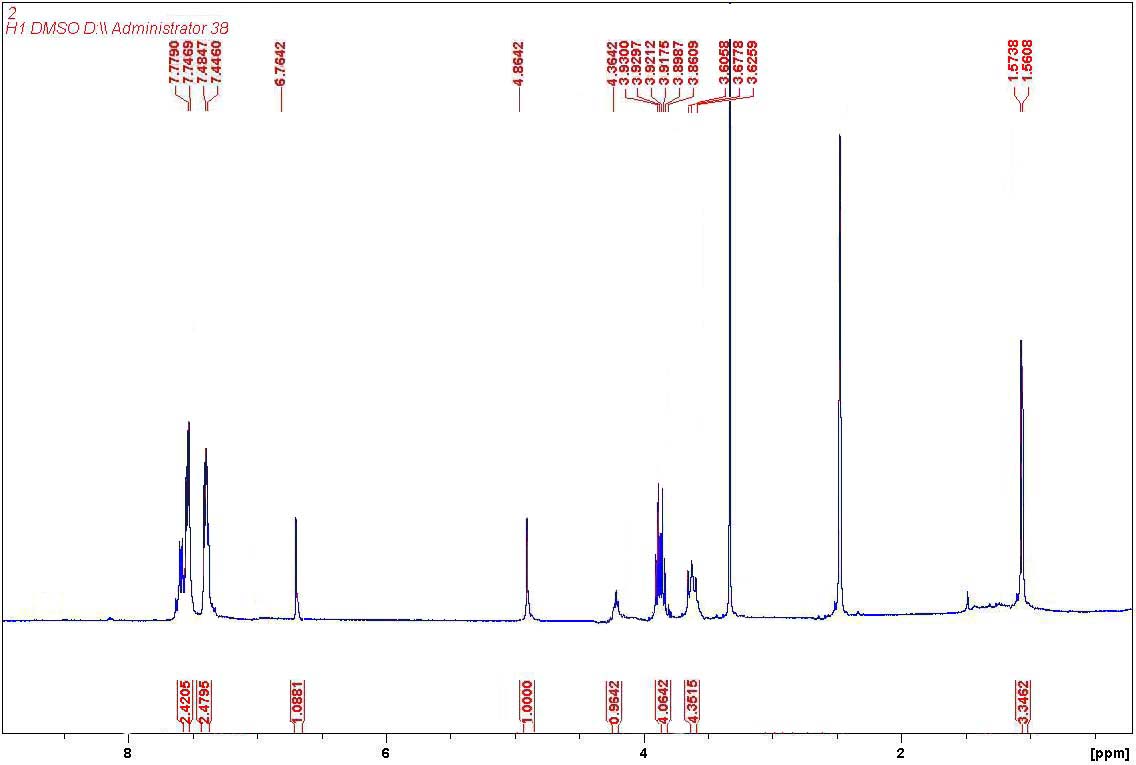


Figure 7. ^1^H NMR spectrum of compound (8h)
